# Increased Posterior Tibial Slope Is Associated with Isolated Meniscal Injuries: A Case-Control Study

**DOI:** 10.3390/medicina61081368

**Published:** 2025-07-29

**Authors:** Kai von Schwarzenberg, Tamara Babasiz, Jan P. Hockmann, Peer Eysel, Jörgen Hoffmann

**Affiliations:** Department of Orthopaedic, Trauma and Plastic Surgery, Faculty of Medicine, University Hospital Cologne, University of Cologne, 50931 Cologne, Germany; tamara.babasiz@uk-koeln.de (T.B.); jan.hockmann@uk-koeln.de (J.P.H.); peer.eysel@uk-koeln.de (P.E.); joergen.hoffmann@uk-koeln.de (J.H.)

**Keywords:** tibial slope, meniscal injuries, meniscal tear, knee joint, risk factor

## Abstract

*Background and Objectives:* The relationship between posterior tibial slope (PTS) and isolated meniscal injuries remains a topic of debate. This study aimed to investigate whether an increased PTS was associated with a higher risk of isolated meniscal tears, using a case-control design with propensity score matching to minimize confounding factors. *Materials and Methods:* A retrospective case-control study was conducted at a University Hospital. A total of 294 patients who underwent arthroscopic surgery for meniscal injuries were compared to a matched control group without documented knee pathology. Two independent observers measured PTS on standardized lateral knee radiographs and assessed inter- and intra-rater reliability. Propensity score matching was performed to control for potential confounders. Statistical analysis included logistic regression to evaluate the association between PTS and isolated meniscal injuries. *Results*: A significantly increased mean PTS was observed in patients with isolated meniscal injuries compared to controls (*p* < 0.05). However, PTS was not significantly associated with the specific location of meniscal tears. Inter- and intra-rater reliability for PTS measurements was excellent (intraclass correlation coefficient > 0.75). *Conclusions*: An increased posterior tibial slope was associated with a higher risk of meniscal injury, even in the absence of ACL rupture. However, no significant association was found between PTS and specific tear patterns or locations. These findings support the role of posterior tibial slope as an independent anatomical risk factor for meniscal damage and underscore the importance of its early identification in clinical risk assessment and prevention strategies.

## 1. Introduction

Meniscal injuries are frequent injuries in athletes, often occurring in conjunction with anterior cruciate ligament (ACL) ruptures during sports activities [1,2]. In addition, degenerative meniscal injuries are a very common cause of knee pain in adults [3]. If not properly addressed, meniscal tears can result in abnormal loading within the tibiofemoral joint, potentially accelerating the wear of the articular cartilage and leading to early onset osteoarthritis [4]. Meniscal tears are influenced by a combination of external conditions and person-specific anatomical and physiological traits. External conditions encompass a variety of factors related to the circumstances under which the injury occurs. These include neuromuscular imbalances that affect joint stability, the nature and direction of forces involved in the injury mechanism, delayed access to medical care, untreated or undiagnosed injuries to surrounding structures such as ligaments, and the way individuals move or land during sports and other physical activities [5,6,7]. In addition to these situational elements, a range of patient-related characteristics can also increase the likelihood of meniscal injury. These include non-modifiable aspects such as age and biological sex, as well as modifiable or measurable traits like limb alignment, an elevated body mass index (BMI), and the morphological configuration of the knee joint—particularly the geometry of the tibial plateau [8,9,10]. Identifying key anatomical elements, like the posterior tibial slope (PTS), can aid orthopedic surgeons in recognizing individuals at greater risk of meniscal tears, enabling more precise prevention and management strategies. The posterior tibial slope (PTS), defined as the posterior inclination of the tibial plateau relative to the tibial shaft axis, is a key anatomical factor influencing tibiofemoral joint mechanics, as it governs the extent of anterior tibial translation [11,12]. An increased PTS increases anterior tibial translation during axial loading, thereby elevating anterior shear forces across the knee joint [13,14]. These forces are transmitted through stabilizing structures, including the meniscus, and may contribute to degenerative changes or acute injury over time, particularly during pivoting or high-impact activities. While the bony geometry itself is non-modifiable, except through invasive osteotomies, recognizing increased PTS early may help guide the implementation of targeted preventive strategies, such as neuromuscular training or load management, to mitigate functional risk. These forces place additional mechanical stress on stabilizing structures, particularly the anterior cruciate ligament and the menisci [15,16]. In ACL-deficient knees, anterior translation is unopposed and contributes to instability; however, even in ACL-intact knees, increased PTS may result in higher strain on the menisci, which act as secondary stabilizers. This repeated mechanical loading—especially during activities involving pivoting, twisting, or repetitive loading—can predispose the menisci to degeneration or acute injury. While PTS itself is a non-modifiable anatomical characteristic except through invasive surgical intervention, its early identification may help guide preventive strategies. These could include neuromuscular training, posterior chain strengthening, or load management approaches aimed at minimizing functional overload on the meniscus in individuals at elevated risk.

Hence, the sagittal plane of the knee is gaining increasing attention. Studies provide evidence supporting the association between increased posterior tibial slope and a higher risk of meniscal injuries, particularly in the presence of ACL injuries [17]. Nevertheless, the evidence for an association between an increased posterior tibial slope (PTS) and meniscal injuries in the absence of ACL injuries is less robust and not as extensively studied as the combined scenario (PTS with ACL injuries). Additionally, studies investigating the aforementioned scenario provide contradictory findings, as some studies identified an increased tibial slope as a risk factor for isolated meniscal injuries [17,18,19], while others find a lower tibial slope as a risk factor for isolated meniscal injuries [20,21]. These conflicting findings highlight a significant gap in the current literature and underscore the need for further clarification.

Therefore, the aim of the present study was to investigate whether an increased posterior tibial slope was associated with an increased risk of isolated meniscal injuries in patients without ACL rupture, and whether it influences the location or pattern of meniscal tears. It was hypothesized that increased posterior tibial slope predisposes to isolated meniscal injury and affects the distribution and morphology of meniscal tears.

## 2. Materials and Methods

This study represents a retrospective analysis of two cohorts, classified as level IV evidence. Ethical approval was granted by the Institutional Review Board of the University of Cologne (ID-number: 24-287533-retro).

### 2.1. Cohort Formation

Patients who underwent arthroscopic meniscus surgery between 1 January 2013, and 31 December 2023, were identified from the digital records of the Department of Orthopaedic and Trauma Surgery at the University Hospital of Cologne. Clinical data relevant to the analysis were extracted for all included patients. In addition to demographic and anthropometric details such as age at the time of surgery, gender, and body mass index (BMI), the precise location of the meniscal lesions as well as the tear type was documented. This describes the morphological classification of meniscal injuries based on the shape and orientation of the tear within the meniscus. Common tear types include horizontal, radial, and bucket-handle tears, each representing a distinct structural pattern with potential biomechanical implications. The lesion location was determined based on arthroscopic findings documented in operative reports. Patients were eligible for inclusion if they had a diagnosed meniscal injury confirmed by arthroscopic findings, with or without concomitant anterior cruciate ligament (ACL) rupture. Exclusion criteria included the presence of concomitant fractures, multiligament injuries, or axial malalignment in the coronal plane (i.e., varus or valgus deformity). Datasets with missing values were excluded from the respective analyses to ensure data integrity.

The control group was derived from radiographic data of patients who presented to the emergency room of the same department within the same time period (January 2013 to December 2023). These individuals underwent knee radiographs due to acute, low-energy blunt knee trauma. Healthy controls were defined as individuals who presented to the emergency department with acute low-energy blunt knee trauma and underwent standard clinical examination and radiographic imaging. Only patients without structural abnormalities on radiographs and without signs or symptoms suggestive of internal derangement—such as joint effusion, locking, ligamentous instability, or focal meniscal tenderness—were included. Patients with known knee pathology or prior history of knee complaints or one or more of the aforementioned symptoms while presenting to the emergency room were excluded. Using propensity score matching, 100 healthy controls were selected based on age, gender, and BMI, with a match tolerance of 0.05 to ensure comparability with the meniscal injury cohort. These controls provided a robust baseline for evaluating the relationship between posterior tibial slope (PTS) and meniscal injuries. The PTS was measured using the two-circle method in both groups.

### 2.2. Radiographic Assessment

The tibial slope was defined to the angle formed by the intersection of the posterior inclination of the tibial plateau and a line perpendicular to the longitudinal axis of the tibial diaphysis. The posterior tibial slope was quantified using lateral knee radiographs and the two-circle method [22].

**Figure 1:**
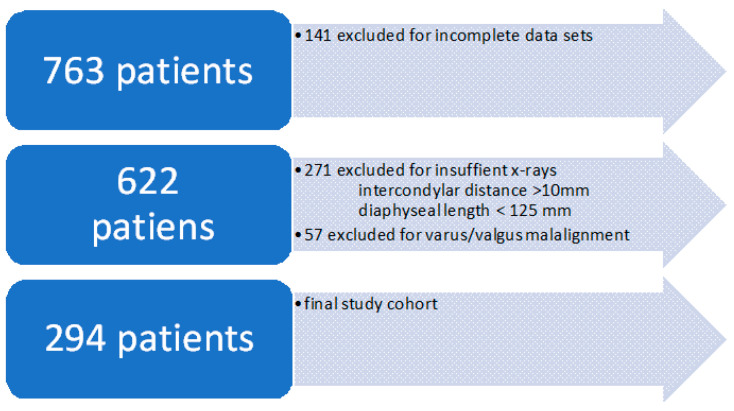
Flowchart of patient exclusion process.

The longitudinal axis of the tibial diaphysis was defined by drawing a vertical line passing through the centers of two circles positioned within the diaphysis: one circle located just below the tibial tuberosity and the other placed distally as far as possible. The medial tibial slope was calculated by measuring the angle formed between a tangent to the medial tibial plateau and a line perpendicular to the established diaphyseal axis (see Figure 1).

To minimize measurement errors associated with rotational malalignment of the knee in radiographs, the intercondylar distance was required to be <10 mm [23]. The posterior intercondylar distance was specifically measured to assess the degree of radiographic malrotation.

Furthermore, a minimum visualized diaphyseal length of 125 mm on the radiograph was required to ensure accurate measurements and reduce error [24]. This criterion was consistently recorded in the study.

Radiographs that failed to meet the specified quality criteria for reliable tibial slope determination led to the exclusion of the respective patients from the analysis. Of the initially identified 763 patients with meniscal injuries, 141 were excluded due to incomplete datasets. Of the remaining 622 patients, 328 were excluded due to substandard radiograph quality, resulting in a final cohort of 294 patients, see Figure 1. The same quality criteria were applied to the control group.

### 2.3. Data Analysis

The radiographs were each measured twice by two independent observers (3rd year resident of orthopedic surgery and orthopedic consultant) using IMPAX EE R20XIX SU1 software (Agfa HealthCare, Mortsel, Belgium), according to the two-circle method in a blinded manner and at an interval of six weeks, and then both the inter-rater correlation coefficient and the intra-rater correlation coefficient were calculated. The inter-rater agreement between the two observers, as well as the intra-rater agreement for both observers for the slope measurement, was calculated using the intraclass correlation (ICC) according to Shrout and Fleiss [25] (absolute agreement). ICC benchmarks were used as proposed by Cicchetti [26] (poor: ICC < 0.4; moderate: 0.4–0.59; good: 0.6–0.74; excellent: 0.75–1.0).

Propensity score matching was performed using 100 healthy individuals matched on age and gender, with a match tolerance of 0.05. Data were presented as median (range) and/or mean ± standard deviation, as appropriate. A *p*-value < 0.05 was considered statistically significant. For regression and correlation analyses, statistical outliers were identified and excluded. Correlations were assessed using Pearson’s correlation coefficient. Although a sample size of *n* > 30 is commonly accepted for assuming normal distribution, normality of the slope was additionally tested using the Kolmogorov-Smirnov and Shapiro-Wilk tests, both indicating a normal distribution. Group comparisons were conducted using one-way ANOVA or Kruskal-Wallis tests, depending on data distribution. G*Power 3 software (Heinrich Heine University, Düsseldorf, Germany) was used for post-hoc power analysis to evaluate the adequacy of the sample size in detecting observed effects. Multiple comparisons were adjusted using the Bonferroni correction.

Graphics were done with GraphPad Prism 9.5.1.

## 3. Results

### 3.1. Patient and Control Group Characteristics

A total of 294 patients with complete radiographs and datasets were included in the analysis. The mean age was 45.1 years (range: 13.77–81.57 years), and females constituted the majority of the cohort at 57.1% (*n* = 168). The mean body mass index (BMI) was 26.08, ranging from 18.59 to 54.69.

The control group comprised 100 matched individuals with a mean age of 42.39 years (range: 14.66–79.97 years). Females represented 63% of this group (*n* = 63). The mean BMI in the control group was 27.13, with values ranging from 17.92 to 51.48, see Table 1.

### 3.2. Meniscal Tear Characteristics

The detected meniscal ruptures were classified into three categories: horizontal tears (*n* = 157), radial tears (*n* = 55), and bucket-handle tears (*n* = 51). In the majority of cases, the medial meniscus was affected (*n* = 199), while in 94 cases, the lateral meniscus was injured.

Regarding medial meniscal injuries, 33 cases involved the anterior horn, 63 cases were in an intermediate location, 163 cases affected the posterior horn, and 2 cases involved a root lesion. Notably, no ramp lesions were detected in any patient (*n* = 0). For lateral meniscal injuries, the anterior horn was affected in 18 cases, the intermediate portion in 29 cases, the posterior horn in 64 cases, and root lesions were identified in 2 cases. In several cases, the rupture incorporated more than only one location of the meniscus.

### 3.3. Posterior Tibial Slope (PTS) Analysis

The average PTS in the study cohort was 7.4 ± 2.8°. The distribution of PTS values varied depending on the location of the meniscal lesion, as presented in Table 2. The PTS values showed a normal distribution.

### 3.4. Test-Retest Reliability

The intra-rater correlation coefficient, i.e., the comparison between the first and second measurement of one observer in each case, was 0.91 for the first observer and 0.79 for the second observer, which is in the excellent range. The inter-rater correlation coefficient, i.e., the comparison between the measurements of both observers, was 0.8, which is also in the excellent range.

### 3.5. Correlations and Statistical Analyses

There was no significant correlation between PTS and age (r(275) = −0.0, *p* = 0.3). Additionally, no significant differences in PTS were observed between different tear types, with horizontal tears averaging 7.3 ± 2.8°, radial tears 7.5 ± 2.9°, and bucket-handle tears 7.3 ± 2.6° (*p* = 0.9).

Among patients with posterior cruciate ligament (PCL) injuries, collateral ligament injuries, or prior meniscal injuries, the average PTS was slightly lower (7.3 ± 2.8°) compared to those without such injuries (7.4 ± 2.8°), although this difference was not statistically significant (*p* = 0.7). However, patients with an anterior cruciate ligament (ACL) injury exhibited a significantly higher PTS (8.0 ± 3.0°) compared to those without an ACL injury (7.2 ± 2.7°, *p* < 0.05).

When comparing meniscal lesion locations, there were no statistically significant differences in PTS values among patients with lateral meniscus injuries (7.5 ± 2.8°), medial meniscus injuries (7.3 ± 2.8°), or injuries involving both menisci (7.6 ± 3.4°, *p* = 0.8). Similarly, patients over the age of 50 years exhibited no significant difference in PTS compared to younger patients (7.4 ± 2.8° vs. 7.4 ± 2.8°, *p* = 0.9).

When evaluating the influence of the surgical approach, patients with sutured meniscal tears had a higher PTS (7.4 ± 2.7°) compared to those who underwent meniscal resection (6.7 ± 2.7°), though this difference was not statistically significant (*p* = 0.2).

**Figure 2:**
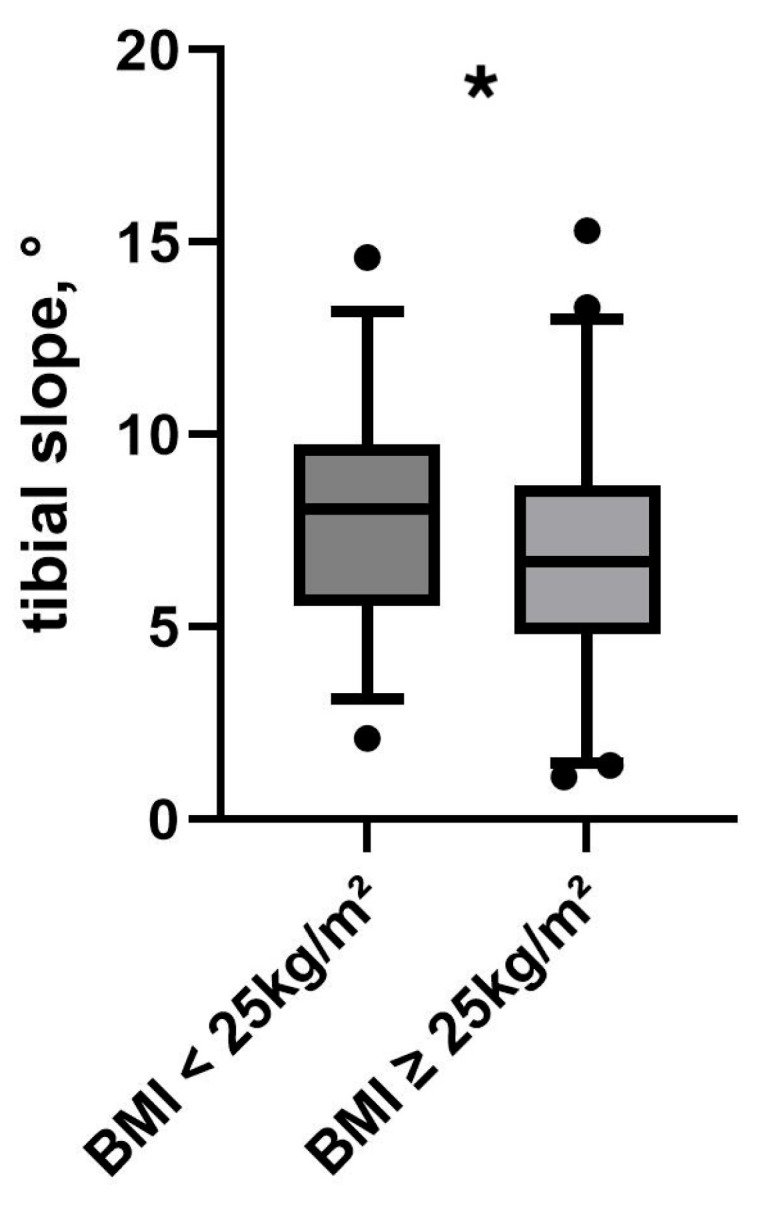
PTS in relation to BMI. Black dots represent outliers. Asterisk (*) indicates a statistically significant difference between groups (*p* < 0.05).

Patients with a normal BMI (18.5–24.9 kg/m^2^) had a significantly higher PTS (7.9 ± 2.9°) compared to overweight patients (7.1 ± 2.8°, *p* < 0.05), see Figure 2. Post-hoc power analysis revealed a statistical power of 0.98, indicating that the study was sufficiently powered to detect the effects under investigation.

### 3.6. Propensity Score Matching Analysis

**Figure 3:**
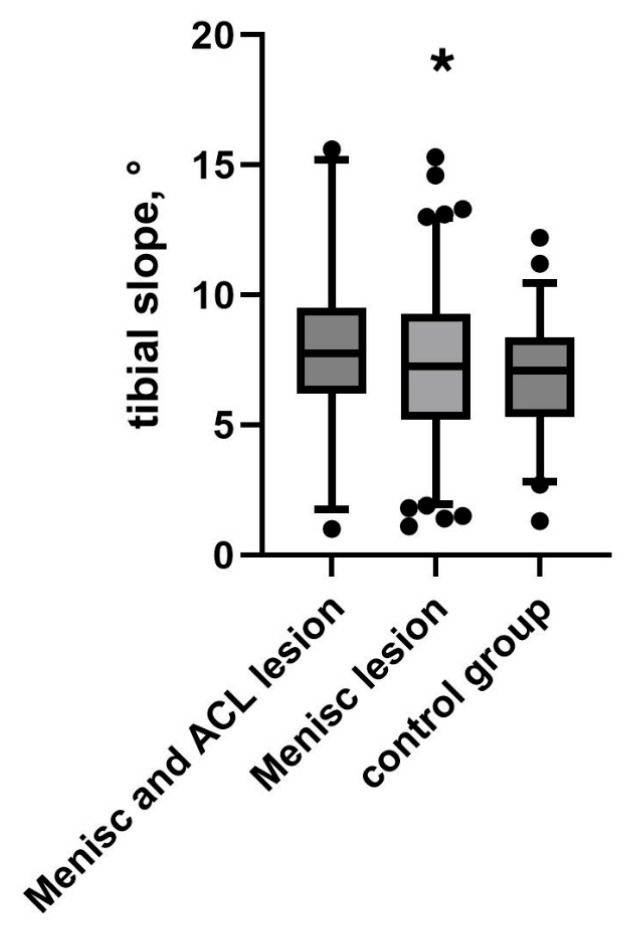
PTS in relation to ACL status compared to healthy controls. Black dots indicate outliers. Asterisk (*) indicates a statistically significant difference between the meniscal lesion group and the control group (*p* < 0.05).

A comparison with 100 healthy controls revealed that patients with meniscal lesions had a significantly higher PTS (7.7 ± 2.8°) compared to healthy individuals (6.8 ± 1.9°, *p* < 0.05). Further analysis compared three groups: patients with both meniscus and ACL lesions (*n* = 72) had a PTS of 8.0 ± 3.0°, those with isolated meniscus lesions (*n* = 222) had a PTS of 7.9 ± 2.9°, and the healthy control group (*n* = 100) exhibited a significantly lower PTS of 6.8 ± 1.9°. The overall difference among these groups was statistically significant (*p* < 0.05), and post-hoc analysis confirmed a significant difference between the meniscus + ACL lesion group and the control group (*p* < 0.05), see Figure 3.

## 4. Discussion

Patients with meniscal lesions had a significantly increased posterior tibial slope than healthy controls. This finding highlights the role of increased posterior tibial slope (PTS) as a biomechanical risk factor for meniscal injuries. It is important to note that the retrospective, case-control design of this study allows only for the identification of associations, not causal relationships. While an increased posterior tibial slope was significantly associated with the presence of isolated meniscal injuries, causality cannot be inferred due to potential residual confounding factors and the absence of longitudinal data. Prospective studies will be necessary to determine whether increased PTS contributes directly to the development of meniscal pathology over time. The relationship between posterior tibial slope (PTS) and meniscal injuries, particularly in the absence of anterior cruciate ligament (ACL) rupture, is a subject of growing interest but remains incompletely understood. While PTS is a well-established risk factor for ACL injuries, which is also reflected in the study, where patients with an ACL injury exhibited a higher PTS compared to those without, its role in isolated meniscal injuries has yielded contradictory findings in existing literature. This study provides new insights by exploring the biomechanical implications of PTS and its association with meniscal injuries, aiming to clarify its significance as a risk factor in the absence of ACL pathology.

The observations of this study suggest that an increased slope alone may create an adverse mechanical environment conducive to meniscal damage. This finding is critical as it demonstrates that PTS may act independently of ligamentous instability to elevate the risk of meniscal pathology.

Although patients with meniscal injuries exhibit increased PTS, no location-specific association between tibial slope and meniscal tear distribution was observed. This aligns with previous research, which has not established a consistent link between tibial slope and specific meniscal injury sites [27,28,29]. This suggests that while PTS contributes to overall meniscal stress, other factors—such as localized joint loading, knee alignment, and individual activity patterns—likely influence the precise location and morphology of meniscal tears.

However, several studies have reported an association between increased PTS and specific meniscal injury sites. Kim et al. found a higher tibial slope in patients with ramp lesions [30], while Okazaki et al., Moon et al. and Kolbe et al. observed an increased tibial slope in patients with medial meniscal posterior root tears [31,32,33]. In the results observed in the presented study, there was no correlation between these injuries and an increased PTS, but the number of patients with these injuries was too small to verify or falsify the hypothesis. Further studies may prove the correlation. Additionally, other studies have linked increased PTS to medial [31,34] and lateral meniscal tears [35]. The variability in reported injury locations across studies suggests that this association is not consistently reproducible across different study populations. These discrepancies may stem from differences in patient populations, imaging methodologies, or variations in biomechanical compensation mechanisms across individuals. Furthermore, most studies identifying these associations examined ACL-deficient knees, which represent a fundamentally different biomechanical state compared to ACL-intact knees.

The observation that a higher PTS was associated with ACL injuries is consistent and extensively studied [36,37,38]. The results of the present study emphasize the interplay between PTS, ACL injuries, and meniscal tears, as patients with ACL injuries in this cohort exhibited significantly higher PTS values than those without, consistent with previous studies. This underscores the critical role of slope in knee joint biomechanics. It suggests that in patients with an increased PTS, even minor trauma or repeated stress can lead to a cascade of injuries, beginning with ACL rupture and progressing to meniscal damage.

The propensity-score matching analysis with healthy controls further underscores the importance of PTS as a risk factor even in ACL-sufficient knees. Patients with meniscal injuries had significantly increased slopes compared to healthy individuals, emphasizing the potential of PTS as a predictive marker for injury risk in the general population. This finding aligns with the hypothesis that PTS amplifies internal knee forces even in stable joints, creating a predisposition to meniscal damage over time or under repetitive stress. Recent biomechanical studies have shown that with increased PTS, the pressure distribution and meniscal forces change in the ACL and PCL sufficient knee [13,39]. These altered biomechanical conditions may predispose for meniscal injury. Even after controlling for overweight as a confounding factor for meniscal tears [40], the findings demonstrate that normal-weight patients (BMI < 25) had significantly increased slopes than both overweight patients and healthy controls. This suggests that the relationship between PTS and meniscal injury risk is not merely an indirect effect of body weight but rather an independent biomechanical factor.

### Clinical Implications

Beyond the biomechanical insights provided by the present findings, the results also carry several noteworthy implications for clinical practice.

Given the association between increased posterior tibial slope (PTS) and isolated meniscal injuries observed in this study, routine measurement of PTS during standard knee radiographs may provide clinically valuable information. In particular, incorporating PTS assessment into the diagnostic workup of patients presenting with unexplained knee pain, recurrent meniscal symptoms, or non-traumatic meniscal lesions could help identify individuals at elevated biomechanical risk. This approach may be especially beneficial in settings where MRI is not immediately available or as part of pre-participation evaluations in athletic populations.

Identifying an increased PTS as an independent risk factor for meniscal injury opens the door for more personalized prevention strategies. In individuals with increased PTS—especially athletes or those engaging in high-load or pivoting activities—targeted interventions such as neuromuscular training, proprioceptive exercises, and strength conditioning could mitigate excessive tibiofemoral loading and reduce injury risk. These non-invasive strategies may be incorporated into rehabilitation protocols or used proactively in sports medicine and orthopedic settings to lower the likelihood of first-time or recurrent meniscal injuries.

The presence of an increased posterior tibial slope has important implications not only for diagnosis and prevention but also for surgical decision-making and postoperative care. In patients undergoing arthroscopic treatment of meniscal lesions, preoperative recognition of an increased PTS may prompt the surgeon to consider the mechanical environment that contributed to the injury. While slope-correcting osteotomies are traditionally indicated for chronic instability or graft failure following anterior cruciate ligament reconstruction, their potential role in patients with recurrent or bilateral meniscal pathology warrants further investigation. Until stronger evidence supports surgical slope correction for isolated meniscal injuries, heightened awareness of PTS can still guide less invasive adaptations in patient management. For instance, rehabilitation protocols may be individualized based on PTS measurements, emphasizing progressive loading and joint stabilization strategies in patients with increased slopes. Furthermore, decisions regarding meniscal repair versus partial meniscectomy may also take into account the underlying tibial slope, given that higher mechanical strain could affect healing potential or accelerate re-injury risk. Finally, these biomechanical considerations may inform long-term surveillance in patients with increased PTS who are at elevated risk for degenerative changes, helping clinicians implement early lifestyle and therapeutic interventions to preserve joint integrity.

## 5. Limitations

This study has several limitations. Its retrospective design limits the ability to establish causality between posterior tibial slope (PTS) and meniscal injuries. As a single-center study, the findings may not be generalizable to broader populations. Additionally, the study focuses on radiographic measurements without longitudinal data or detailed clinical outcomes, making it difficult to assess the functional impact of PTS. The inclusion of only surgically treated cases may introduce selection bias, as conservatively managed meniscal injuries were not considered. Furthermore, important contextual factors such as the mechanism of injury—whether sports-related, traumatic, or degenerative—as well as behavioral and lifestyle-related influences like physical activity level, sports participation, and occupational knee loading were not systematically recorded and therefore could not be included in the analysis. The absence of this information limits the ability to interpret the findings in relation to specific risk profiles and reduces the generalizability of the results to defined populations, such as athletes versus non-athletes. Although care was taken to select controls from the same time frame as the injury group, the retrospective nature of the data collection may still introduce a risk of timing bias, which could affect the comparability of exposure or diagnostic conditions over the study period.

## 6. Conclusions

This study confirms a significant association between increased posterior tibial slope (PTS) and the presence of isolated meniscal injuries, supporting the hypothesis that PTS is an independent anatomical risk factor even in the absence of ligamentous instability. Furthermore, an increased PTS was associated with concomitant ACL injuries, reinforcing prior evidence of shared biomechanical vulnerability. However, no significant differences in PTS were observed across meniscal tear locations or patterns, suggesting that increased PTS may predispose to meniscal injury in a more general biomechanical manner, rather than influencing specific injury types. These findings underscore the relevance of PTS as a non-modifiable risk factor and highlight the potential value of its early identification in clinical risk stratification and prevention strategies.

## Figures and Tables

**Table 1:** Demographic characteristics.

Demographic Characteristics	Cohort	Control Group
Age, mean (min–max) years	45.34 (13.77–81.57)	42.39 (14.66–79.97)
Sex, *n* (%)		
Male	126 (42.86)	37 (37)
Female	168 (57.14)	63 (63)
BodyMassIndex of the cohort, mean (min–max) kg/m^2^	26.08 (18.59–54.69)	27.13 (17.92–51.48)

**Table 2:** PTS values for the different locations.

Location of the Lesion	Slope °
Medial meniscus (*n* = 199)	7.39 ± 2.85
Anterior horn (*n* = 33)	7.01 ± 2.68
Intermedial (*n* = 63)	7.38 ± 2.87
Posterior horn (*n* = 164)	7.45 ± 2.86
Root lesion (*n* = 2)	7.95 ± 1.77
Lateral meniscus (*n* = 79)	7.54 ± 2.92
Anterior horn (*n* = 18)	8.62 ± 3.12
Intermedial (*n* = 29)	6.42 ± 2.6
Posterior horn (*n* = 64)	7.43 ± 2.8
Root lesion (*n* = 2)	9.4 ± 2.69

## Data Availability

The original contributions presented in this study are included in the article. Further inquiries can be directed to the corresponding author.
